# Mitochondrial Dynamics in Blood Cancer Development and Progression

**DOI:** 10.1007/s40495-025-00431-0

**Published:** 2025-10-25

**Authors:** Saurav Doshi, Christina Glytsou

**Affiliations:** 1 Graduate Program in Pharmaceutical Sciences, School of Graduate Studies, Rutgers University, Piscataway, 08854 NJ USA; 2 Department of Chemical Biology, Ernest Mario School of Pharmacy, Rutgers University, Piscataway, 08854 NJ USA; 3 Department of Pediatrics, Robert Wood Johnson Medical School, Rutgers University, New Brunswick, 08901 NJ USA; 4 Rutgers Cancer Institute of New Jersey, New Brunswick, 08901 NJ USA; 5 Susan Lehman Cullman Laboratory for Cancer Research, 164 Frelinghuysen Rd, Room 115, Piscataway, 08854 NJ USA

**Keywords:** Fusion, Fission, Mitochondria, Blood cancer, Leukemia

## Abstract

**Purpose of Review:**

This article outlines the role of mitochondrial dynamics in healthy cells and elaborates on how blood cancer cells hijack these processes to support uncontrolled proliferation, stemness, and drug resistance. A comprehensive understanding of the mechanistic details of mitochondrial behavior in malignant hematopoiesis will provide new therapeutic avenues and improve the prediction of therapy responses.

**Recent Findings:**

Mitochondrial dynamics, governed by the complementary events of fusion and fission, is a key cellular process for maintaining metabolic flexibility, organelle integrity, and cellular homeostasis. Impairment of the dynamic fusion-fission balance can lead to various chronic pathologies. Recent research has highlighted how blood cancer cells exploit mitochondrial remodeling to maintain metabolic efficiency and adjust organellar quality control mechanisms to sustain survival pathways and enable cancer progression. Furthermore, leukemia and lymphoma cells use mitochondrial plasticity to adapt under stress conditions and to evade cell death induced by various clinically used or tested therapeutic regimens. Investigations using blood cancer cell lines, patient-derived samples, and xenograft models have begun to uncover the specific roles and regulatory mechanisms of mitochondrial dynamics proteins in different subtypes of hematologic malignancies, as well as in therapy resistance. Additionally, preclinical studies suggest that targeting these regulators may present novel therapeutic opportunities and serve as predictive biomarkers in blood cancers.

**Summary:**

This review highlights the therapeutic potential of modulating mitochondrial dynamics, underscoring the need for further integrative studies to fully harness this vulnerability in hematologic malignancies.

## Introduction to Mitochondrial Characteristics

Mitochondria, theorized to have originated from the initial endosymbiotic alphaproteobacterial ancestor equipped with genomic replication and conjugation capabilities, have since evolved to adapt to the cytosolic environment of the eukaryotic host [1]. This symbiosis is believed to have conferred a survival advantage to the host cell in an oxygen-rich environment. Over time, the majority of the bacterial DNA was transferred to the host cell’s nucleus to protect it from toxic metabolites produced by the mitochondria, establishing nuclear control over the organelle [2].

Since the initial discovery of mitochondria as the powerhouse of eukaryotic cells, our understanding of their diverse roles has greatly expanded. In addition to generating cellular energy through oxidative phosphorylation (OXPHOS), mitochondria are involved in cell metabolism, programmed cell death, autophagy, signaling, redox balance, innate immunity, stem cell reprogramming, and calcium homeostasis [3–7].

Mitochondria can engage in these multiple cellular processes due to their unique compartmentalization. Structurally, a mitochondrion is a bi-membraned organelle consisting of the outer mitochondrial membrane (OMM) and the inner mitochondrial membrane (IMM).

The OMM acts as an interface between the cytosol and the mitochondrial lumen. The OMM hosts a repertoire of proteins, such as translocases mediating the transport of mitochondrial precursor proteins, ion channels, and proteins that coordinate organelle crosstalk [8]. This allows mitochondria to establish membrane contacts with the endoplasmic reticulum (ER), as well as lysosomes, peroxisomes, endosomes, melanosomes, lipid droplets, and the plasma membrane [9]. The OMM also contains proteins involved in a wide range of cell signaling pathways, allowing mitochondria to serve as a signaling hub [7, 10, 11].

The IMM surrounds the mitochondrial lumen and is folded into pleomorphic invaginations known as cristae. The cristae increase the surface area, allowing maximum space to accommodate the mitochondrial respiratory chain complexes and the F_1_F_o_-ATP synthase. This enhances the production of ATP through the OXPHOS. Further, the intracristae compartment confines various soluble metabolites, such as cytochrome *c* in the cristae lumen, which can be released into the cytosol in response to specific stimuli [9, 12].

## Mitochondrial Dynamics

**Fig. 1:**
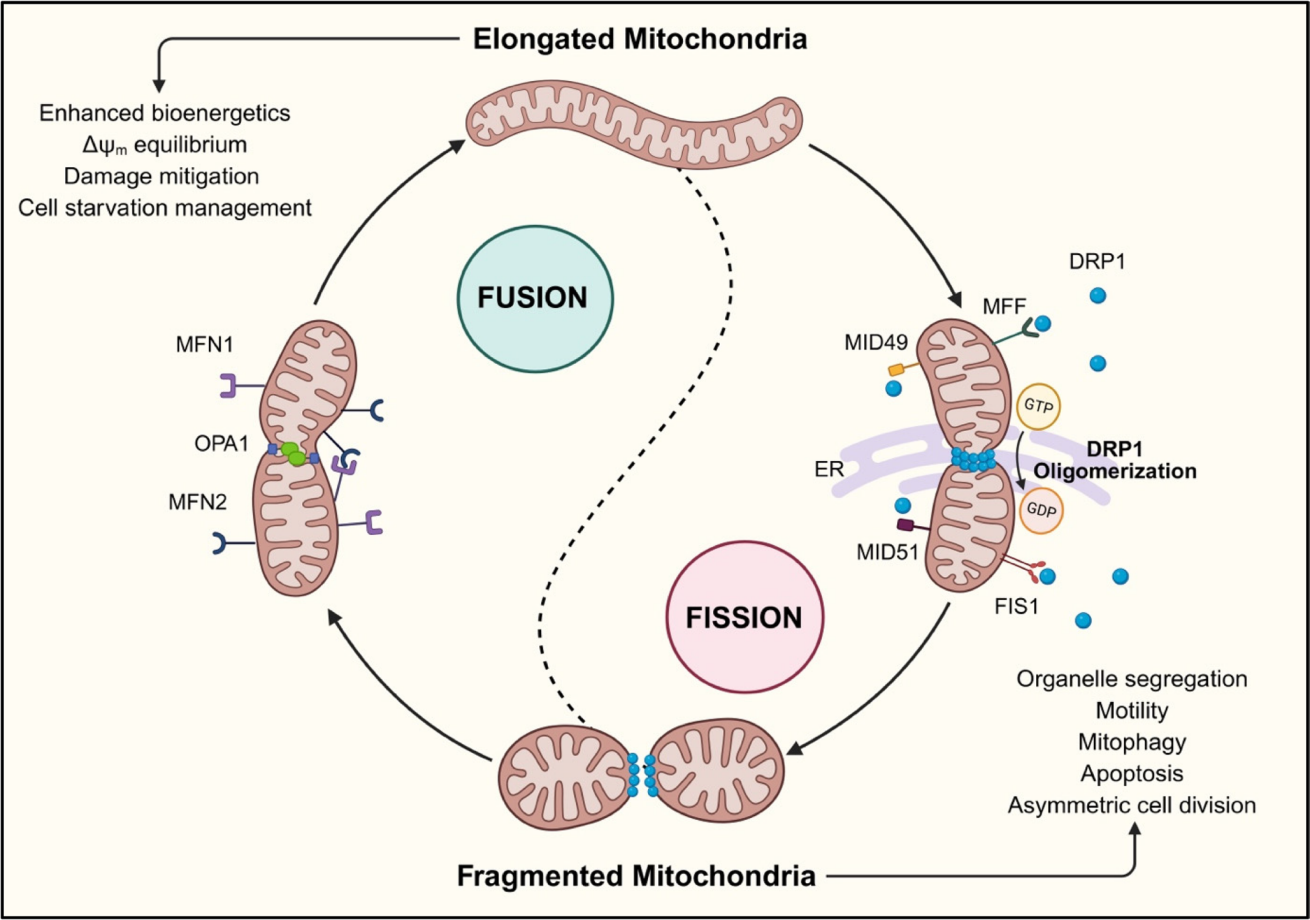
Mitochondrial dynamics: Fusion and Fission; a yin and yang representation. Mitochondria undergo fission via DRP1 oligomerization catalyzed by GTP hydrolysis. Mitochondrial-ER interaction sites mark the location of fragmentation. DRP1 interacts with FIS1, MFF, MID49, and MID51, which are present on the OMM to accomplish fission. MFN1/2 on the OMM and OPA1 in the mitochondrial inter-membrane space achieve mitochondrial fusion. The balance between these processes is critical to maintain cellular homeostasis (Figure was created in https://BioRender.com). ER: endoplasmic reticulum; OMM: outer mitochondrial membrane; IMM: inner mitochondrial membrane

Initially considered as structurally static organelles, mitochondria are now known to have a dynamic morphology and ultrastructure regulated by mitochondria-shaping proteins. Multiple studies have highlighted the importance of mitochondrial flexibility in adapting to physiological cellular cues through its dynamic architecture. Mitochondria can exist either as isolated organelles or connected in networks, unevenly distributed to meet the local cellular energy needs. This adaptability of mitochondria, involving changes in morphology, subcellular interactions, and cytosolic localization, is referred to as mitochondrial dynamics [13]. The two main mechanisms of mitochondrial membrane dynamics include fusion and fission. This mitochondrial yin and yang balance is crucial for maintaining normal mitochondrial function and cellular homeostasis (Fig. 1). Interestingly, these mechanisms are not only governed structurally by mitochondrial proteins and lipids but also by cytosolic signals in response to cues such as nutrient starvation [12].

###  Key Players of Mitochondrial Fusion

Mitochondrial fusion involves the close contact of two mitochondria, followed first by the OMM and then IMM fusion. Fusion is primarily governed by three dynamin-like proteins (DLPs); mitofusin 1 (MFN1), mitofusin 2 (MFN2), and optic atrophy 1 (OPA1), all of which are GTPases. MFN1 and MFN2 are located on the OMM [14]. While genetically and structurally homologous, MFN1 and MFN2 play distinct functions [15]. MFN1 interacts with OPA1 to initiate mitochondrial fusion [16], whereas MFN2 is involved in mitochondrion–mitochondrion interactions to form mitochondrial clusters and interactions between mitochondria and the ER [17, 18].

OPA1 has a bifunctional role in mitochondrial dynamics. It is involved in OMM and IMM fusion and the shaping of cristae [16, 19]. OPA1 is subject to post-translational modifications, mainly due to proteolytic cleavage by YME1L and OMA1 peptidases, resulting in the formation of long (L-OPA1) and short (S-OPA1) forms of OPA1 [20, 21]. L-OPA1 is anchored into the IMM, while S-OPA1 is peripherally associated with the membrane facing the intermembrane space. Under normal conditions, L-OPA1 and S-OPA1 coexist as oligomers. A study proposes that fusion depends on the appropriate balance between L-OPA1 and S-OPA1 [22]. Based on work by Chan’s laboratory, a combination of both L-OPA1 and S-OPA1 forms boosts IMM fusion, while OXPHOS stimulates the controlled OPA1 proteolytic cleavage by YME1L, which mediates mitochondrial fusion [22, 23]. A more recent study proposed that the stoichiometry between L-OPA1 and S-OPA1 is necessary for the complete and efficient IMM fusion [24]. On the other hand, other in vitro studies revealed that L-OPA1 establishes two types of interactions between membranes; homotypic trans interactions between two L-OPA1 molecules that are fusion-incompetent and heterotypic trans interactions between L-OPA1 and cardiolipin, which are IMM fusion-competent. The addition of S-OPA1 to the trans L-OPA1 and cardiolipin complex enhances membrane fusion.

With regards to the cristae formation, it is proposed that L-OPA1 homotypic interactions lead to the tethering of adjacent cristae membranes, maintaining tight cristae junctions [25]. OPA1 expression is directly correlated with cristae width [19, 26]. In addition, OPA1 interacts with multiple IMM-residing proteins, including MIC60 and ATAD3A, to form high-molecular-weight complexes that control mitochondrial ultrastructure [27, 28]. Notably, OPA1 also regulates apoptotic cristae remodelling, which is a striking reorganization of the IMM, including the inversion of cristae curvature with simultaneous mobilization of the cytochrome *c* enclosed within the cristae [3, 19]. Upon an apoptotic stimulus, OPA1 undergoes excessive proteolytic cleavage, leading to the dissociation of OPA1-containing complexes, a concomitant loss of mitochondrial cristae integrity, and complete cytochrome *c* release, ultimately amplifying the apoptotic pathway [19, 27, 29].

### Key Players of Fission Machinery

Mitochondrial fission is the division of the organelle into two separate entities. The master regulator of mitochondrial division is the dynamin-1-like protein (DRP1) encoded by the *DNM1L* gene [30]. DRP1 is a GTPase that predominantly localizes in the cytosol. Upon a stimulus, DRP1 translocates onto the mitochondria, where it binds to OMM protein adaptors, including mitochondrial fission factor (MFF), mitochondrial dynamics proteins 49 and 51 (MID49 and MID51), and mitochondrial fission 1 (FIS1). DRP1 activity is tightly regulated by various post-translational modifications, including phosphorylation, ubiquitination, sumoylation, O-GlcNAcylation, and S-nitrosylation [31–35]. The phosphorylation status of DRP1 dictates its recruitment to the mitochondria [36, 37]. Cryo-EM analysis has revealed that DRP1 oligomerizes and forms a complex with GTP, which is stabilized by the OMM proteins. This binding, paired with GTP hydrolysis, gives rise to DRP1-mediated membrane constriction, ultimately causing mitochondrial membrane fission [38]. Mitochondria-ER contact sites regulate mitochondrial division by defining the sites where DRP1 will be recruited and assembled [39]. Notably, Myosin II and OMM-recruited actin are also involved in the oligomerization and activity of translocated DRP1. OMM constriction is mediated by myosin motor proteins and the actin cytoskeleton. Fission cannot occur or can be reverted in the absence of actin polymerization, which is required to exert membrane tension during fission. As a general mechanism, at ER-mitochondria contact sites, ER-bound Inverted Formin 2 (INF2) nucleates actin, which provides the platform for DRP1 recruitment and oligomerization of DRP1 to constriction zones. Also, microtubules modulate DRP1 recruitment to mitochondria, thereby shifting mitochondrial morphology toward fragmentation [40]. There, DRP1 binds to actin, and the latter promotes DRP1 GTPase activity. The DRP1-actin binding dynamics are governed by GDP and GTP, which accelerate and decelerate it, respectively. Additionally, MFF, MID49, MID50, and FIS1 can influence the DRP1-actin interaction and the GTP hydrolysis [41–44]. Besides actin and myosin, mitochondrial homeostasis and dynamics are governed by kinesin and dynein moving along the tubulin filaments. Various microtubule-based motor proteins influence the movement of DRP1 from the cytosol to mitochondria. Kinesin-3 promotes mitochondrial fission by activating calcineurin, which dephosphorylates DRP1 at Serine 637, leading to DRP1 translocation to mitochondria [45]. In contrast, under oxidative stress, Kinesin-1 enhances DRP1 phosphorylation at Serine 616, which also promotes DRP1 recruitment to mitochondria. The dynein-dynactin complex also mediates DRP1 recruitment to the mitochondria [46]. Mitochondria bind to myosin XIX, kinesin-1, and dynein-dynactin through the adaptor proteins - GTPases Miro1 or Miro2 (collectively called Miro1/2) [40, 47–49]. Besides controlling mitochondrial trafficking within cells, Miro1/2 negatively regulates DRP1 recruitment to mitochondria, thereby suppressing fission in a calcium-dependent manner [50, 51].

Both fusion and fission proteins have multiple functions and interacting partners that can be context-specific. Interestingly, overexpression of either MFN2 or FIS1 proteins has been associated with mitochondrial fragmentation. In the case of FIS1, this is owing to excessive recruitment of DRP1 to mitochondria, which induces fission. On the other hand, an excess of MFN2 in cells with wild-type MFN1 can lead to mitochondrial clustering, decreased mitochondrial membrane potential, and organelle deformation, hinting that a stoichiometric imbalance between the key fusion and fission regulators can yield mitochondrial dysfunction and a punctuate network [18, 52]. Also, unlike MFN1, MFN2 deficiency in cardiomyocytes leads to enlarged mitochondria in cardiomyocytes, possibly due to the considerable differences between MFN2 and MFN1 GTPase activity in heart tissue [53]. Under physiological conditions, the expression levels of fusion and fission machinery are finely regulated, yet in pathological states, whether experimentally induced or disease/stress-driven, the balance can be disrupted, leading to mitochondrial fragmentation or dysfunction.

### Fusion Promotion or Fission Inhibition in Healthy Cells

Fission and fusion are vital tasks in the mitochondrial life cycle. These transitions between elongated and fragmented mitochondrial states help maintain a healthy mitochondrial population by eliminating damaged material [54]. Additionally, mitochondrial dynamics are detrimental to balancing energy demand, nutrient supply, and mitochondrial quality control. Favoring connected or fragmented mitochondrial architecture influences bioenergetic efficiency, energy expenditure, and cell fate.

Fusion, along with increased cristae abundance, is positively associated with higher bioenergetic efficacy and enhanced ATP production [9, 12]. Overexpression of OPA1 promotes the assembly and efficiency of respiratory supercomplexes by tightening mitochondrial cristae, thereby enhancing OXPHOS efficiency [26]. Conversely, the deletion of OPA1, MFN1, or MFN2 leads to decreased oxygen consumption [26, 55]. Fusion has also been observed during the G_o_, G_1,_ and G_1_-S phases of the cell cycle in rat kidney cells. The G_0_ phase is characterized by both filamentous and fragmented mitochondria. Mitochondria convert from a fragmented, isolated state into a hyperfused giant network at G_1_-S transition. The network is electrically continuous and has greater ATP output than mitochondria at any other cell cycle stage. In G1-S, most surprisingly, mitochondria form a giant tubular network, with tubular elements undergoing fission and fusion. Similar cell cycle mitochondrial phenotypes are seen in synchronized cells released from G_0_ by relief from serum starvation, with fragmented/intermediate phenotypes in G_1_ shifting to tubular in G_1_-S, and back to fragmented/intermediate in S and G_2_-M [56]. Another key role of mitochondrial fusion is acting as an organelle quality control mechanism, thus helping cells cope with various insults. Mitochondria in starved cells stay in the elongated state longer by inhibiting DRP1 recruitment, preventing their clearance via autophagy, allowing highly efficient OXPHOS, and sustaining cell viability [4, 57]. Mild metabolic stress induces hyperfusion [58]. Complementation and sharing of mitochondrial DNA (mtDNA) and its products during fusion can rescue mitochondrial dysfunction caused by mtDNA mutations [59]. Similarly, fusion-mediated component exchange supports the membrane potential equilibrium and controls signaling events mediated via calcium oscillations [54, 60]. The relevance of fusion for organismal health is further substantiated by the embryonic lethality observed in mouse models ubiquitously lacking *Mfn1*, *Mfn2*, or *Opa1* [61, 62].

Mitochondrial fusion is exercised in various cell types during normal functioning. Hematopoietic stem cells (HSCs) in mice have longer mitochondria and express higher levels of *Mfn2* transcript and protein relative to more mature populations, while MFN2 is essential in maintaining HSCs with lymphoid potential through regulation of calcium signaling [63]. Memory T cells, which need to be in a catabolic state for prolonged survival, tend to have fused mitochondria. This elongation facilitates fatty acid distribution and oxidation within the mitochondrial network, increasing their spare respiratory capacity [64, 65]. Likewise, regulatory T (T-reg) cells display fused mitochondria that reprogram their metabolism to support differentiation, fatty acid oxidation and enhance ATP production for immunosuppressive function. A major role of transforming growth factor beta 1 (TGF-1β) is the activation of mitochondrial fusion in Treg cells [66]. Furthermore, murine mesenchymal stem cells (MSCs) shift from fission to fusion by upregulating *Mfn1* or *Mfn2* to achieve differentiation into adipocytes and osteocytes [67]. Elongated mitochondria are also observed in senescent cells through the downregulation of FIS1 and DRP1, acting as a quality control mechanism against stress-induced organelle damage [68–70]. In tissues, like skeletal muscle of mice, fusion is necessary to maintain mtDNA integrity, protecting against lethal mitochondrial genome mutations [55]. Another study in rats showed that mitochondrial fusion preserves cellular bioenergetics and excitation-contraction coupling in skeletal muscle fibers [60]. Furthermore, using a mitofusin knockout mouse model and embryonic stem cells with lower *Mfn2* and *Opa1* levels, Kasahara and colleagues demonstrated that mitochondrial elongation drives calcium and Notch1-dependent cell signaling to support mesodermal cell differentiation into cardiomyocytes [6]. Table 1 summarizes the different mitochondrial dynamics trends observed in healthy cells.

**Table 1:** Association of mitochondrial morphological States with functional outcomes across healthy cell types

Cell type	Mitochondrial dynamics state (Key regulators)	Functional outcome	Reference
Hematopoietic stem cells	Fusion/elongation (MFN2)	Maintain lymphoid potential via Ca²⁺ signaling.	[63]
Mesenchymal stem cells	1.Shift to fusion (MFN1/MFN2 increase) 2. Shift to fission	1. Enables differentiation to adipocyte/osteocyte. 2. Favors chondroblastic differentiation.	[67]
Breast tissue stem like cells	Fission	Enables asymmetric division	[71]
Memory T cells	Fusion/elongation	Fatty acid oxidation and increased spare respiratory capacity for long-term survival.	[64, 65]
Regulatory T cells	Fusion/elongation (TGF-β1 driven)	Reprograms metabolism toward fatty acid oxidation to support immunosuppression.	[66]
Effector T cells	Fission/fragmentation	Supports anabolic, short-lived effector state.	[64]
Migrating T-lymphocytes	Fission with edgeward trafficking	Positions mitochondria at leading edge for local ATP.	[72]
Senescent cells	Fusion/elongation (FIS1/DRP1 decrease)	Protects against stress-induced organelle damage.	[69]
Skeletal muscle fibers	Fusion frequent	Supports excitation–contraction coupling.	[60]
Cardiomyocytes	Fusion/elongation	Drives cardiomyocyte differentiation via calcineurin/Notch1 signaling.	[6]
Neonatal cardiomyocytes (heart development)	Fission-regulated mtDNA nucleoid dynamics	Maintains uniformly active mitochondria during neonatal heart development.	[73]
Kidney cells	Fusion	Maintains a transient hyperfused mitochondrial network for regulating G1-S phase transition.	[56]

### Shifting Toward Fission in Healthy Cells

Cells exposed to a nutrient-rich environment retain the fragmented mitochondrial state to balance nutrient utilization with the ATP demand. Mitochondrial fission decreases bioenergetic efficiency as a protective mechanism against the harmful effects of nutrient overload, diverting energy from nutrient oxidation toward heat production. This shift results in OXPHOS impairment, creating an energy sink that does not involve ATP synthesis, while increasing mitochondrial surface area and proton conductance [74].

Moreover, mitochondrial fission mediates organelle segregation, generating daughter mitochondria associated with different levels of membrane potential (ΔΨm); one organelle with a higher ΔΨm and another with a lower ΔΨm. Mitochondria with lower ΔΨm can either recover their membrane potential and regain the capacity to reconnect with the network or remain depolarized. If the membrane potential is not restored during the solitary depolarized period, OPA1 is degraded, fusion is inhibited, and the organelle will be cleared by selective autophagy (mitophagy). Therefore, fission is an important quality control mechanism for isolating and eliminating damaged organelles [54, 75].

When severe damage triggers the initiation of the intrinsic pathway of apoptosis, mitochondria fragment in a DRP1-dependent manner [76]. This morphological alteration is accompanied by OMM permeabilization and apoptotic cristae remodeling, allowing the complete cytochrome *c* release into the cytosol, which amplifies the apoptotic cascade [3, 77].

Mitochondrial fission is also required during asymmetric cell division, as it ensures the dispersal of old and young mitochondria between daughter cells, maintaining stemness and pluripotency [71]. Additionally, the distribution of mitochondrial DNA (mtDNA) during cell division is also ensured by fission [78]. Furthermore, mitochondrial division mediates intracellular organelle motility and distribution to allow localized energy expenditure, interactions with other organelles, and cell signaling [79].

Different cells utilize mitochondrial fission to achieve different cellular goals. Mitochondrial autophagy, which can be regulated by the peroxisome proliferator-activated receptor d (PPAR-δ) fatty-acid oxidation (FAO) pathway, is required for the maintenance of murine HSCs [80, 81]. Therefore, ongoing DRP1 activity and balanced mitochondrial dynamics are presumably essential for effective mitophagy and, thus, the control of asymmetric HSC cell division and HSC quiescence [82]. Interestingly, during active replication, HSCs accumulate dysfunctional mitochondria due to the inactivation of DRP1 and the decrease in mitochondrial fission, driving the loss of HSC regenerative potential and HSC attrition. Genetic or pharmacologic targeting of DRP1 in mice leads to HSC pool expansion, but it causes a functional decline of the progeny after replication stress [83]. These findings suggest that fine-tuning mitochondrial dynamics may also have therapeutic applications for preserving HSC functionality. Unlike memory T cells, effector T cells exhibit higher mitochondrial fission to achieve an anabolic state with smaller mitochondria scattered throughout the cytoplasm [64]. T-lymphocytes also use fission to allow mitochondria distribution to the “leading edges”, providing local ATP necessary for cell movement and migration [72]. Interestingly, unlike MSCs differentiating into adipocytes, the ones differentiating into chondroblasts favor mitochondrial fission [67]. Mitochondrial fragmentation also supports organelle trafficking to the neuronal synapses to accommodate high local energy demands and calcium handling [84, 85]. Murine neonatal cardiomyocytes utilize DRP1-mediated mitochondrial fission to maintain proper mtDNA nucleoids dynamics and thus a homogeneous active mitochondrial distribution during heart development [73] (Table 1).

### Fusion and Fission in Disease

As mitochondrial dynamics play a vital part in cellular homeostasis, an imbalance between fusion and fission processes has been associated with various diseases. Mutations in fusion/fission factors cause human pathologies. Specifically, MFN2 mutations are linked to Charcot-Marie-Tooth disease (CMT) and OPA1 mutations are associated with autosomal dominant optic atrophy (ADOA) [86–88]. DRP1 and MFF mutations have also been connected to encephalopathies [89, 90]. Dysfunction in mitochondrial dynamics has also been observed in chronic renal diseases [91], neurodegenerative disorders like Parkinson’s, Alzheimer’s, and Huntington’s disease, diabetes, cardiovascular diseases, and cancer [92–97]. A bird’s-eye view of mitochondrial dynamics involvement in crucial elements of cancer pathogenesis, such as uncontrolled cell proliferation, immune surveillance, drug resistance, and metastasis, has been previously provided (reviewed in Corrado et al. 2012 [98]; Ma et al. 2020 [99]; Kumar et al. 2022 [100]; Chen et al. 2023 [101]).

Our review sheds light on the relevance of mitochondrial dynamics dysfunction in hematologic malignancies. It delves into the mitochondrial fusion-fission imbalance in the different subsets of blood cancer, underlining the current knowledge gaps. By illuminating these areas, we aim to provide a deeper understanding of how mitochondrial dynamics influence the pathogenesis and progression of hematologic malignancies, as well as therapy resistance, identifying potential therapeutic strategies for future research.

## Why is Mitochondrial Dynamics Crucial in Cancer Progression?

The transformation of normal to malignant tumor cells is a complex, multistep process. During tumorigenesis, cells undergo several structural and functional changes at the molecular and cellular level called “hallmarks of cancer” [102]. Alterations such as cancer-driving mutations, cytogenetic aberrations, epigenetic changes, and post-translational modifications result in abnormal transcription, channel function, signaling pathways, and metabolic routes, promoting persistent cell proliferation and resistance to programmed cell death [103].

Metabolic reprogramming is a well-established hallmark of cancer, first described by Otto Warburg in 1923 [104]. In his landmark study, as described by Racker, Warburg observed that cancer cells exhibit predominantly aerobic glycolysis rather than oxidative respiration, a phenomenon known as the Warburg effect [105]. While initially thought to be a universal trait of malignant cells, a plethora of recent studies have now demonstrated that tumors display a more flexible balance between cytoplasmic and mitochondrial ATP production [106, 107]. This phenomenon underscores the critical role of mitochondrial functions in both tumorigenesis and responses to anti-cancer therapy (reviewed in Di Gregorio et al. 2022 [108]).

But how do mitochondrial dynamics influence cancer progression? Recent research has provided shreds of evidence describing the implication of mitochondrial dynamics in various steps of tumor development, from initiation to progression, in different human cancers. Fusion and fission allow mitochondria to dynamically adjust their morphology in response to the changing cellular energy demands, enabling cancer cells to adapt to metabolic stress, make energy adjustments, and support the rapid proliferation of cancer cells (reviewed in Liu et al. 2023 [109]).

Mitochondrial dynamics influence core cancer hallmarks by rewiring metabolism, survival, proliferation, invasion, and therapy response. Table 2 summarizes how different mitochondrial dynamics regulators support these hallmarks in different cancers. On the fission side, DRP1-driven fragmentation is positioned as a lever that shapes disease behavior. Increased mitochondrial division, driven by the upregulation of DRP1, has been observed in various solid cancers, including lung cancer, breast cancer, colon cancer, glioblastoma, melanoma, and renal carcinoma [120, 141–144]. In these cancers, the occurrence of mitochondrial fission has been correlated with metabolic reprogramming, cell cycle progression, increased migration, invasiveness, metastatic potential, and chemoresistance. DRP1-targeting using the Mitochondrial division inhibitor 1 (Mdivi-1) resensitizes chemoresistant hepatocellular carcinoma, lung, and breast cancer cell lines to cytotoxic cisplatin treatment [145]. Fragmented, DRP1-high networks in latent breast cancer brain tropic cells create “mitochondrial puncta” that favor fatty-acid oxidation (FAO) to sustain bioenergetics and redox control. Genetic *Drp1* depletion or the brain-penetrant DRP1 inhibitor Mdivi-1 reduces lipid use, increases lipid-droplet accumulation, and attenuates brain metastatic burden in vivo. Additionally, inhibiting DRP1 or restoring fusion suppresses growth and promotes apoptosis in multiple models, underscoring dynamics as an upstream governor of cell-cycle and cell death [101]. Higher DRP1 and lower MFN1 levels facilitate mitochondrial division and recruitment to lamellipodia regions, hence promoting breast cancer cell migration during metastasis to lymph nodes [118]. In the context of mitochondria and cytoskeleton interaction in cancer, Miro proteins and cytoskeletal elements also work in parallel with DRP1, where mitochondria are recruited to the leading edge. Migrating tumor cells concentrate mitochondria at the leading edge and require local, mitochondria-derived ATP to build lamellipodia [146]. In invasive breast cancer lines, this correlates with a fission bias where DRP1 is upregulated. Either DRP1 knockdown or MFN1 (fusion) overexpression suppresses lamellipodia formation, linking pro-migratory behavior to the fusion-fission balance [118]. Cytoskeletal inputs tune that balance at multiple steps. In melanoma, the mitochondria-anchored isoform of the actin nucleator SPIRE1 (mitoSPIRE) recruits myosin MYOVA to be placed adjacent to DRP1 to facilitate fission [147]. Within this circuitry, Miro GTPases act as cytoskeletal dynamics hubs, negatively regulating DRP1 recruitment. Loss of Miro GTPases mispositions mitochondria and impairs collective migration [148]. In epithelial and fibroblast models, AMP-activated protein kinase (AMPK) senses local ATP depletion and drives mitochondrial relocalization to the leading edge, further coupling energy cues to mitochondrial dynamics and motility [146]. Destabilization of Miro1-TRAK2 transport complex produces perinuclear mitochondrial clustering, which is expected to alter fusion-fission homeostasis during invasion in the tumor stroma [149]. Such studies link fission-driven plasticity to the hallmarks of metabolic reprogramming, metastasis, and death-evasion hallmarks [101, 119].

**Table 2:** Functional roles of mitochondrial dynamics proteins in supporting the cancer hallmarks

Mitochondrial dynamics regulator	Cancer type	Hallmark of cancer	Reference
DRP1	AML	Resisting therapy-induced cell death	[110, 111]
	CML	Sustaining proliferative signaling, and resisting cell death	[112, 113]
	Lymphoma	Resisting cell death	[114]
	T-ALL	Resisting cell death	[115]
	Multiple myeloma	Therapy resistance	[116, 117]
	Breast cancer	Activating invasion and metastasis	[118, 119]
	Lung cancer	Sustained cancer proliferation	[120]
	Hepatocellular carcinoma	Tumor cell survival	[121]
FIS1	AML	Enabling stemness and resisting cell death	[122–124]
MFF	AML	Resisting cell death	[125]
MARCH5	AML	Sustaining proliferative signaling, resisting therapy-induced cell death	[126–128]
MFN1	AML	Resisting cell death	[129]
	Cholangiocarcinoma	Tumor proliferation	[130]
	Multiple myeloma	Sustaining proliferative signaling and resisting cell death	[131]
MFN2	AML	Resisting therapy-induced cell death	[132–135]
	T-ALL	Resisting therapy-induced cell death	[136]
OPA1	AML	Resisting therapy-induced cell death and supporting proliferation and bioenergetics.	[126, 128, 137]
	Cholangiocarcinoma	Tumor proliferation	[130]
	Melanoma	Angiogenesis	[138]
OMA1	Lymphoma	Therapy resistance	[139]
CLPB	AML	Resisting therapy-induced cell death and supporting proliferation and bioenergetics	[140]

Conversely, mitochondrial fusion proteins MFN1, MFN2, and OPA1 have also been associated with tumor progression. In hepatocellular carcinoma and cholangiocarcinoma, MFN1- and OPA1-mediated fusion is excessively activated, while the deletion of these genes impairs mitochondrial bioenergetics and inhibits cell growth [130]. Elevated OPA1 levels correlate with poor prognosis in breast cancer and non-small cell lung cancer [150–152]. In KRas-mutant lung adenocarcinoma, OPA1 plays critical roles in mitochondrial respiration and NAD + regeneration through regulating cristae morphology [153]. Metastatic breast cancer cells upregulate OPA1 and depend on it for respiration, migration, and proliferation. Genetic OPA1 loss or novel small-molecule OPA1 inhibitors such as MYLS22 and its second-generation derivative Opitor-0 selectively blunt metastatic-cell proliferation and migration without comparable effects on non-metastatic counterparts [154]. In addition, due to OPA1’s anti-apoptotic function, its targeting using MYLS22 or Opitor-0 synergizes with the BCL-2 inhibitor ABT-737 in paclitaxel-resistant breast cancer cell lines [155]. Similarly, OPA1 targeting increases gefitinib-induced cell death in lung adenocarcinoma and sorafenib-induced apoptosis in liver cancer [151, 156, 157]. Interestingly, OPA1 has also been identified as a critical regulator of tumor angiogenesis [138]. Lastly, *MFN2* can act as either a tumor suppressor or an oncogene in cancer progression, depending on the context [158]. Collectively, these findings link mitochondrial membrane architecture and fusion to the cancer hallmarks of sustaining cellular energetics and proliferative signaling, resisting cell death, activating metastasis, and inducing angiogenesis.

Changes in mitochondrial morphology are also associated with reactive oxygen species (ROS) generation. Increased mitochondrial fission, due to elevated DRP1 to MFN1 ratio, causes higher ROS production, which supports cancer survival in hepatocellular carcinoma [121]. In turn, ROS can modulate mediators of both mitochondrial fusion and fission in a dose-dependent manner, thereby promoting cancer cell migration and chemoresistance (reviewed in Brillo et al. 2021 and Kim et al. 2016 [159, 160]). Cancer cells activate Ras/MAPK/ERK, PI3K/Akt, p53, and NF-κB pathways through ROS, further enhancing cell survival, apoptosis resistance, and metastasis (reviewed in Prasad et al. 2017) [161]. However, excessive ROS accumulation can induce oxidative stress, causing nuclear DNA damage and activating DNA damage responses [162]. It can also lead to mtDNA lesions and potential mtDNA degradation [163]. Furthermore, mitochondrial dynamics can also serve as a quality control mechanism in cancer cells. Fission can isolate and eliminate abnormal mitochondrial fragments through mitophagy, limiting ROS overproduction. Conversely, fusion can help mitigate mitochondrial dysfunction by buffering mitochondrial ROS production, preserving mitochondrial membrane potential, and minimizing mitochondrial damage [162, 164]. These adaptive responses are crucial for protecting cancer cells against proteotoxic stress and the hallmark of maintaining their survival under hostile conditions.

Mechanistically, there is a direct governance of critical cancer signaling pathways over mitochondrial dynamics. Under energy stress, AMPK directly drives fission by phosphorylating the DRP1 receptor MFF, increasing DRP1 recruitment to mitochondria. In parallel, AMPK initiates autophagy/mitophagy by phosphorylating Unc-51-like Autophagy Activating Kinase 1 (ULK1) and suppresses mTORC1 through phosphorylation of Tuberous Sclerosis 2 (TSC2) and Regulatory-Associated Protein of mTOR (RAPTOR), thereby de-repressing autophagy transcriptional programs and reshaping metabolic signaling [165]. By contrast, when tumor cells are exposed to PI3K inhibitors, a paradoxical reactivation of AKT1/2 and mTOR emerges, which repositions elongated, respiration-competent mitochondria to the cortical cytoskeleton, where they fuel lamellipodia dynamics, accelerate focal adhesion turnover, and increase migration and invasion [166]. Therefore, AMPK-coupled fission, mitophagy, and mTOR downregulation push cells toward catabolic, quality control programs, whereas PI3K-Akt-mTOR-driven mitochondrial relocalization hardwires energy supply to the cell edges for motility.

Recent studies pinpoint the role of mitochondrial dynamics alterations in anti-tumor immunity. Enhanced mitochondrial fission via DRP1 in hepatocellular carcinoma leads to mitochondrial stress and mtDNA release into the cytosol. Since mtDNA acts as a damage-associated molecular pattern (DAMPs), this triggers inflammatory responses and causes the secretion of CCL2. Subsequently, this leads to macrophage recruitment and polarization and thus tumor promotion [167]. This highlights the role of mitochondrial dynamics in aiding the hallmark of tumor-promoting inflammation.

Recent computational analyses and high-throughput drug screenings revealed that altered mitochondrial dynamics can exhibit differential anti-cancer drug sensitivities [168]. For example, knocking out *OPA1* and *DNML1* sensitizes cell lines of ovarian carcinoma, melanoma, breast cancer, lung cancer, and pancreatic cancer to the second mitochondrial-derived activator of caspases (SMAC) mimetics; LCL161, BV6, and Birinipant, which block the inhibitors of apoptosis (IAP) family of proteins. Interestingly, breast and lung cancer cell lines without amplification of mitochondrial dynamic genes are insensitive to SMAC mimetics. Further supporting the concept of differential drug sensitivity, the study also showed that *OPA1* or *DNML1* deletion in the MCAS ovarian carcinoma cell line causes sensitization to BCL-2 and MCL-1 inhibitors but not to apoptosis-inducing etoposide [168]. Together, these studies suggested that perturbation of the mitochondrial dynamics network promotes targetable vulnerabilities across different tumors. Further research is required to fully understand the exact roles of fusion and fission machinery in various cancers and therapy responses.

## Mitochondrial Dynamics in Blood Cancer

### Acute Myeloid Leukemia (AML)

AML is a hematopoietic neoplasm involving the abnormal accumulation and proliferation of immature myeloid progenitor cells in the bone marrow. It is a heterogeneous clonal stem cell disorder at both clinical and biological levels. The disease has poor clinical outcomes and high mortality, with an overall five-year survival rate of 15% to 30% [169, 170]. Diagnostic classification of AML strides from seven French-American-British (FAB) classification subtypes to a more sophisticated classification based on cytogenetic abnormalities, gene expression, and mutations [171].

AML cells have increased mitochondrial mass and predominantly rely on oxidative phosphorylation for their survival and stemness [172, 173]. Recent studies uncovered the critical roles of mitochondrial dynamics regulators in AML progression and therapy responsiveness. FIS1 is notably upregulated in AML patient samples, particularly in the leukemic stem cell (LSC) population. High FIS1 activity is a crucial requirement for LSCs to sustain elevated mitophagy rates, preserving their stemness, while FIS1 loss attenuates mitochondrial autophagy, impairing AML stem and progenitor potential [122]. With regards to how mitochondrial fission is regulated in AML LSCs, Pei and colleagues identified AMPK as a master upstream regulator of FIS1 in AML [122, 174]. In further support of these findings, FIS1 expression positively correlates with BM blast counts and leukemic burden at initial diagnosis, as well as poorer therapeutic outcomes [123, 124]. Like FIS1, additional studies suggest that mitochondrial fission factor MFF holds prognostic relevance in AML. Likewise, inhibition of the mitochondrial division regulators, MFF or DRP1, exhibits an anti-leukemic effect in genetically engineered murine models by disrupting OXPHOS. MFF depletion also causes the accumulation of damaged mitochondria through dysregulated mitophagy [125]. In the same study, a DRP1 inhibitor (Mitochondrial division inhibitor 1, Mdivi-1) was used; despite showing an antiproliferative effect in vitro in two AML cell lines, its efficacy was not reflected in vivo in THP-1 transplanted xenografts [125].

Mitochondrial fusion has also emerged as a vulnerability in AML. The genetic deletion of *MFN2* or *OPA1* in AML cell lines and patient-derived xenografts reduces AML viability and initiation. Mechanistically, Larue et al. suggested that suppressing mitochondrial fusion disrupts respiration, inhibits ROS production, and leads to cell cycle arrest in AML [175]. However, in another study, while Kinoshita et al. also reported that loss of OPA1 results in G1 cell cycle arrest in AML cell lines, they showed increased ROS production in these cells, highlighting the need for further investigation [125]. Additionally, targeting MARCH5, a mitochondrial E3 Ubiquitin ligase, which regulates various mitochondrial dynamics proteins (MFN2, DRP1) [176, 177], appeared to curtail AML cell proliferation by inducing mitochondrial fragmentation [126–128]. Furthermore, MFN2 plays a crucial role in AML by regulating mitochondria-ER interactions. Loss of MFN2 disrupts mitochondrial-ER associated membranes (MAMs) in AML cells, hence impairing autophagosome formation, lipid catabolism, FAO, and OXPHOS, while also preventing the elimination of defective mitochondria through mitophagy [127, 132].

Therapy resistance remains a significant challenge for the treatment of AML, with mitochondrial structure and dynamics playing vital roles in its development. A genome-wide loss-of-function CRISPR/Cas9 screen performed in the MOLM-13 AML cell line identified that mitochondrial organization proteins act as synthetic lethal targets when combined with the new AML medication, the BH3 mimetic and potent BCL-2 antagonist - Venetoclax. Among the top hits was a mitochondrial chaperonin, CLPB, which was shown to control mitochondrial ultrastructure maintenance. CLPB depletion leads to loss of normal cristae structures, increased susceptibility to apoptotic cristae remodeling, cytochrome *c* release, and, ultimately, apoptosis. Moreover, these studies uncovered that CLPB interacts with OPA1, regulating its proteolytic processing, though the precise mechanistic details of CLPB-OPA1 interplay are still poorly understood. Notably, CLPB and OPA1 are overexpressed and stabilized in Venetoclax-resistant AML cells, emphasizing the key role of mitochondrial structure in the acquisition of drug resistance [137, 140]. From a translational perspective, both the novel OPA1 inhibitors MYLS22 and Opitor-0 reverse the mitochondrial ultrastructural and metabolic adaptations that drive resistance to BH3 mimetics in AML. These small molecules restore apoptotic sensitivity in AML patient-derived xenografts (PDXs), both ex vivo and in vivo. Furthermore, biochemical assays using these OPA1 inhibitors provided insights into new vulnerabilities in glutamine metabolism, proposing new combination strategies - not only with Venetoclax, but also with ferroptosis-inducing agents and glutaminase inhibitors [137].

Another recent study, combining CRISPR/Cas9 loss-of-function screens, super-resolution microscopy, and biochemistry, pinpointed MFN2 and MARCH5 as primary modes of AML resistance to BH3 mimetic drugs [127]. The findings demonstrated increased mitochondrial fusion in resistant cells, which may act as a quality control mechanism to buffer the drug-induced organelle damage and resist apoptotic fragmentation. In addition, elevated MFN2 levels expand MAMs surfaces, which serve as platforms for autophagosome formation and facilitate mitophagy activation upon an insult [133]. MFN2 also recruits the PARKIN protein to initiate the autophagic clearance of “injured” mitochondria following sublethal BH3 mimetic treatment [134]. Hence, high MFN2 abundance in resistant mitochondria enlarges their surface area and increases the number of receptors available for instantaneous mitophagy initiation, helping cells withstand therapeutic stress. Importantly, newly developed mitofusins inhibitors (MFI8) enhance the killing efficacy of apoptosis-inducing agents in AML PDXs [127, 135]. Overall, these studies place mitochondrial dynamics alongside BCL-2-regulated apoptosis as an axis for therapy resistance in AML.

Meanwhile, additional reports highlighted additional mechanisms of mitochondrial dynamics contributing to resistance in AML. Research using the human AML cell lines HL60 and THP-1, as well as the murine AML model expressing the *MLL-AF9* fusion gene, revealed that interleukin 6 (IL6) promotes chemoresistance by enhancing MFN1-mediated mitochondrial fusion and boosting OXPHOS [129].

On the other hand, targeting mitochondrial fission has also shown therapeutic promise in preclinical models. One study proposed that inhibiting DRP1-mediated fission with the compound Mdivi-1 increases Venetoclax-induced apoptosis in the *TP53* mutant AML cell lines (THP-1 and Kasumi-1) [110]. Furthermore, Borthakur and colleagues reported that indirect inactivation of DRP1 through ERK1/2 inhibitors impairs mitochondrial fragmentation, leading to a significant loss of mitochondrial membrane potential and hypersensitivity to Venetoclax in a relapsed AML PDX [178]. In summary, as highlighted by a clinically oriented review by Iyer et al. 2024, mitochondrial alterations in acute leukemia, including changes in membrane potential, metabolism, architecture, and dynamics, play a central role in supporting leukemic survival and therapy resistance [179].

### Chronic Myeloid Leukemia (CML)

CML is a myeloproliferative disorder caused by the chromosomal translocation t(9;22)(q34;q11) in a hematopoietic stem cell. This drives the expansion of a leukemic clone via expression of the fused protein BCR-ABL1 [180]. This disease mostly affects older adults with an average age of 66 years and comes with a good prognosis. The recent 5-year survival rate for CML (2015–2021) is 70.4% [181].

Previous studies have demonstrated that, like AML LSCs, CML LSCs also rely on OXPHOS for their survival [182]. However, the implication of mitochondrial dynamics in CML pathogenesis has not been extensively investigated. Only recently, a report provided evidence that BCR-ABL1 induces mitochondrial fragmentation in CML. Mechanistically, Suzuki et al. proposed that this excessive fission is controlled by MAPK signaling via increasing DRP1 phosphorylation and activation. Treatment with high doses of Mdivi-1 reduces cell viability by inducing necrosis in CML cells [112]. Differentiation inhibition factor 3 (DIF3) is a morphogen that exhibits anti-leukemic efficacy against leukemia cell lines, however, the mechanism of action is not clear. A report using the CML cell line, K652 and CD34 + cells isolated from CML patients indicated that DIF3 efficiently kills cells through the induction of DRP1-mediated mitochondrial fission [113].

### Multiple Myeloma (MM)

MM is a plasma cell dyscrasia involving the clonal proliferation of antibody-producing plasma cells [183]. These abnormal plasma cells release a large amount of a single type of nonfunctional antibody known as paraprotein. The survival rate for myeloma is 62.4% [181]. A recent report identified mitochondrial biogenesis gene signatures upregulated in MM compared to normal plasma cells. At the same time, the expression of a few of them, including MFN1, positively correlates with poor prognosis and inferior patient survival [131]. Another study demonstrated the influence of AMPK-related protein kinase 5 (ARK5) protein on mitochondrial morphology in human myeloma cells KMS-11 and Sachi. ARK5 shifts the dynamic balance towards mitochondrial fission. *ARK5* knockout results in an increased level of MFN1, MFN2, and OPA1, and decreased serine 616 phosphorylation of DRP1 in MM cells. These alterations correlate with elevated mitochondrial ROS and lactate production in ARK5-deleted MM cells [184]. Also, a work focusing on the activity of flavanones Nargenin and Hesperitin reveals that these natural products inhibit DRP1 activity in a human MM cell line NCI-H929, suppressing OXPHOS, triggering ER-stress apoptotic responses while reducing the activity of master transcriptional factors c-myc and SREBF-1. These effects of Nargenin and Hesperitin might explain their activity in decreasing the clonogenicity and viability of MM cells [116]. Similarly, Zhang and colleagues recently proposed that the cholesterol-lowering medication lomitapide acts as an anti-MM agent, overcoming bortezomib resistance by inducing excessive DRP1-mediated mitophagy and, subsequently, mitochondrial damage [117].

Furthermore, a very recent study shed some light on the role of mitochondrial dynamics in the bone marrow niche of MM. Komemi and colleagues profiled bone-marrow mesenchymal stem cells from myeloma niches (MM-MSCs) and showed they maintain MM survival through increased mitochondrial elongation, stress-coping bioenergetics and enhanced spare respiratory capacity (SRC). Pushing the network away from fusion using the OPA1 inhibitor MYLS22 reduces mitochondrial membrane potential, perturbs MM-MSC glycolysis/OXPHOS coupling, and lowers mitochondrial ROS compared to the normal donor MSCs, weakening their pro-tumor support. Using co-culture systems, the authors also demonstrated that low doses of Venetoclax can depress mitochondrial SRC in MM-MSCs and sensitize co-cultured MM cells to bortezomib, positioning mitochondrial respiratory capacity and fusion as selective stromal targets to overcome drug resistance in myeloma [185].

### Acute Lymphocytic Leukemia (ALL)

Acute lymphoblastic leukemia, also referred to as acute lymphocytic leukemia, is characterized by the uncontrolled growth of lymphoid cells arrested at an early stage of differentiation. ALL is the most common pediatric cancer, accounting for 20% of all childhood cancers [186]. The 5-year survival rate of ALL is 72.6% [181]. There is a primary categorization of ALL cases into either B-cell ALL (B-ALL) or T-cell ALL (T-ALL). B-cell malignancies comprise most human blood cancers and represent the most common types of lymphoid tumors [187]. Specifically, for B-ALL, intensive multi-agent chemotherapy has achieved long-term cure in ≥ 90% of children, whereas the overall cure rate is around 50% in adult patients [186].

Transmission electron microscopy image analysis indicated that mitochondria from B-ALL patient lymphoblasts have a more compact and condensed cristae structure than healthy lymphocytes. These shape alterations correlate with increased mitochondrial biogenesis, potentially supporting the bioenergetic needs of B-ALL cells [188]. A more mechanistic study showed that the vital survival regulator in B lymphocytes, TRAF3, interacts with MFF on mitochondria, affecting their post-translational modifications and inducing mitochondrial fragmentation and apoptosis. This study was performed on resting spleen B cells; however, it highlights the relevance of targeting mitochondrial fission and MFF in B cell malignancies [189]. Noteworthy, Venetoclax-resistant B-cell precursor ALL PDXs show larger elongated mitochondria, further supporting the correlation between altered mitochondrial structure, augmented mitochondrial metabolism, and BH3 mimetics resistance in blood cancers [190].

With regards to T-ALL, a study reported that Jurkat cells and primary T-ALL cells in contact with mesenchymal stem cells show a mitochondrial morphology shift from an elongated to a fragmented state. Cai and colleagues showed that this occurs due to the phosphorylation of DRP1 on serine 616, allowing T-ALL cells to achieve chemoresistance and metabolic reprogramming [115]. Conversely, Decker and colleagues demonstrated that MFN2-induced mitochondrial fusion is vital for Jurkat cell survival, chemoresistance, and insensitivity post-doxorubicin treatment [136].

The contrasting roles of DRP1 in ALL subtypes underscore the highly context-dependent nature of mitochondrial fission in acute leukemia. In T-ALL, microenvironmental cues, like mesenchymal stromal interactions, trigger DRP1 phosphorylation, mitochondrial fragmentation, and chemoresistance. Conversely, in B-ALL, mitochondrial fission can be pro-apoptotic, especially in cases of Venetoclax resistance. These opposing outcomes highlight that fission may support survival in T-ALL while promoting apoptosis in B-ALL. As a result, whether DRP1 inhibition helps or hurts depends on lineage, microenvironmental context, and treatment status. Therefore, a uniform approach to therapeutically modulate fission is unlikely to be effective across all ALL subtypes.

### Chronic Lymphocytic Leukemia (CLL)

Chronic lymphocytic leukemia (CLL) is a slow-growing lymphoproliferative disorder characterized by the aberrant accumulation of monoclonal B-lymphocytes in the peripheral blood [191]. When a B-lymphocyte population is detected in enlarged lymph nodes of patients without peripheral lymphocytes, the condition is referred as small lymphocytic lymphoma (SLL), indicating a clinical variant of the same histopathological and molecular entity [192]. CLL is a common leukemia affecting adults, with a 5-year survival rate of 88.8% [181]. Despite this, most patients experience relapse and require further treatment. Similar to AML, Venetoclax therapy is becoming the first-line treatment for CLL and SLL as well due to the observed universal BCL-2 overexpression in these blood cancers [193–195]. Other approved therapies for treating CLL include tyrosine kinase inhibitors, monoclonal antibodies, and nucleoside analogues [196].

CLL cells exhibit higher total mitochondrial content and mitochondrial respiration rates compared to normal B-lymphocytes, leading to elevated mitochondrial ROS. This increased oxidative stress is associated with chemoresistance in this malignancy [197]. As CLL cells depend highly on mitochondria for survival, targeting mitochondria bioenergetics may elicit promising results in CLL treatment.

Additionally, clonal shifts and heterogeneous evolutionary trajectories are observed in CLL patients who relapse upon Venetoclax-based therapies. A small subset of CLL patients displayed a mutation in the Venetoclax binding site of BCL-2 [198]. Furthermore, MCL-1 overexpression and increased OXPHOS activity have been determined as mechanisms for resistance to BCL-2 inhibition in CLL cells [199]. In spite of the emerging significance of mitochondrial reprogramming in CLL and targeted therapy resistance, mitochondrial dynamics remain unexplored.

### Lymphoma

Lymphoma represents a heterogeneous group of malignant neoplasms of lymphocytes, which can involve lymphatic tissue, bone marrow, or extranodal sites. It is traditionally classified broadly as non-Hodgkin or Hodgkin lymphoma, with over 90 subtypes. The initial stratification is derived from B-cell, T-cell, or natural killer cell origin, with further classification based on morphology, immunophenotype, genetic, molecular, and clinical features [200]. The 5-year survival rate for Hodgkin’s lymphoma is 89%, and 74.2% for non-Hodgkin’s lymphoma [181]. Other commonly known lymphoma subtypes in non-Hodgkin lymphoma are Diffuse Large B-Cell Lymphoma (DLBCL) and follicular lymphoma, with a 5-year survival rate of 64.8% and 84% respectively [181].

Recent research has highlighted the critical role of mitochondrial function and biogenesis in B-cell lymphomas. A recent study showed that germinal‐center (GC) B cells dramatically remodel their mitochondria and ramp up mitochondrial protein synthesis as they enter the GC program​ [201]. Using animal models, the authors also proved that deleting the mitochondrial transcription factor TFAM in B cells prevents GC formation and significantly reduces lymphoma formation. This occurs as the high rates of mitochondrial translation enabled by *Tfam* expression are essential for developing B-cell lymphoma. Previously, Caro and colleagues identified two major subclasses of DLBCL; the OXPHOS-DLBCL subtype and the BCR-DLBCL subtype [202]. The OXPHOS-DLBCL subtype is characterized by an enrichment in mitochondrial gene signatures. OXPHOS‐DLBCLs have a distinct metabolic profile and display enhanced mitochondrial energy transduction, tricarboxylic acid cycle activity, fatty acid oxidation, and glutathione synthesis. In contrast, the BCR-DLBCL subtype has a high BCR signaling activity, displays a BCR signaling gene signature, and relies more on glycolysis than mitochondrial respiration. Furthermore, Krug and colleagues recently demonstrated that angioimmunoblastic T-cell lymphoma (AITL) is also marked by OXPHOS gene signatures when comparing patient tumor tissues with healthy lymph nodes [203]. They verified that AITL cells heavily rely on mitochondrial respiration for their survival. Altogether, these data indicate that enhanced mitochondrial biogenesis and metabolic activity help drive lymphoma development, particularly in certain subtypes, while mitochondrial‐high phenotypes appear to have a growth advantage in the hypoxic, nutrient‐limited tumor environment.

Mitochondrial metabolic and structural states shape lymphoma cell survival and responsiveness to therapy. Tumors that maintain high mitochondrial function or evade mitochondrial apoptosis tend to resist conventional treatments. Therapy resistance due to mitochondria is also evident in DLBCL, wherein the OXPHOS-DLBCL subtype, which relies on mitochondrial bioenergetics, shows insensitivity to BCR signaling inhibition. Moreover, a study using six DLBCL cell lines found that the fission protein DRP1 is required for BAX-mediated cell death. Specifically, when DRP1 is knocked down, UV irradiation fails to drive BAX onto the mitochondria, thereby blocking apoptosis ​[114].

Therapeutic strategies to indirectly target mitochondrial dynamics using novel activators of the mitochondrial stress protease OMA1 have also been explored in DLBCL. OMA1 activation causes excessive proteolytic cleavage of OPA1 and increased integrated stress response (ISR)-induced apoptosis in lymphoma cells [139]. More specifically, BTM pyrazolo-thiazoles (BTM-3528 and BTM-3566) selectively kill lymphoma cells by allosterically turning on OMA1, which then cleaves its two key substrates, OPA1 and DELE1, to couple mitochondrial dynamics to the ISR and intrinsic apoptosis. In lymphoma cell lines like BJAB and HCT-116, BTM compounds cause OMA1-dependent loss of long OPA1, inducing fission, without the immediate mitochondrial depolarization or acute respiration impairment. This indicates a specific OMA1 engagement rather than a general bioenergetic collapse upon treatment with these compounds. In addition, BTM-3566 triggers OMA1-mediated DELE1 cleavage, which activates HRI, leading to eIF2α phosphorylation and ATF4 induction. These compounds show a potent and selective anti-lymphoma activity, favorable pharmacokinetics, and in vivo efficacy in PDX models. Importantly, considering that BTM-induced apoptosis is BAX dependent, these candidates can be paired with BCL-2 inhibitors like Venetoclax and likely synergize in specific malignancies. However, their effectiveness is tumor-specific and may be limited by resistance mechanisms driven by upregulation of negative regulators such as FAM210B protein in lymphoma [139].

These findings emphasize the importance of mitochondrial dynamics in lymphoma progression and the development of new therapeutic approaches. Given the selective nature of current therapeutic candidates, metabolic stratification of lymphoma, such as classification into OXPHOS and BCR DLBCL subtypes, can sharpen patient selection and therapy matching, improve clinical utility by serving as predictive biomarkers, and accelerate translational adoption of mitochondrial-targeted strategies.

## Conclusions

**Fig. 2:**
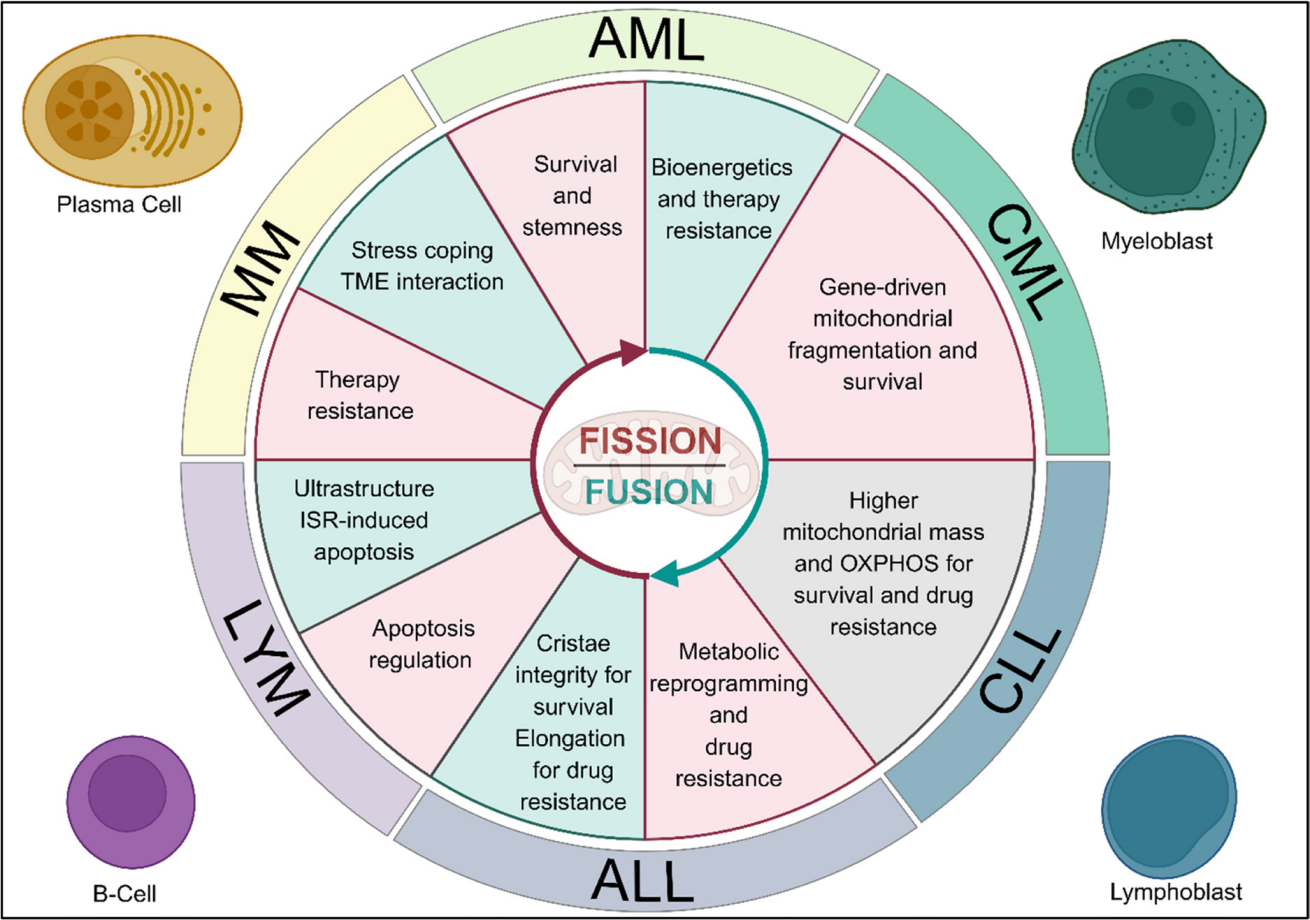
A graphical summary of evidence for the significance of mitochondrial dynamics in cancer survival, proliferation, and drug resistance in various blood cancers. Zones highlighted in red and blue represent cellular events downstream of fission and fusion, respectively. While some studies in CLL have focused on the role of mitochondria in cancers, mitochondrial dynamics remains less explored. This is signified by the grey zone (Figure was created in https://BioRender.com). AML: acute myeloid leukemia; CML: chronic myeloid leukemia; CLL: chronic lymphocytic leukemia; ALL: acute lymphocytic leukemia; LYM: lymphoma; MM: multiple myeloma; OXPHOS: oxidative phosphorylation; ISR: integrated stress responses; TME: tumor microenvironment

With the advent of new fundamental knowledge on mitochondrial dynamics machineries, advanced imaging technologies, and the increasing availability of blood cancer models, our understanding of the various roles of mitochondrial fusion and fission in hematologic malignancies has significantly deepened. Emerging evidence underscores the critical contribution of mitochondrial morphological adaptations in the development, progression, and therapy resistance of blood cancer. In this review, we describe the various downstream effects of altered mitochondrial dynamics on cancer cell fate, including enhanced energy production, metabolic reprogramming, ROS buffering, organelle clearance, elimination of mitochondrial damage, modulation of cell signaling pathways, and apoptosis evasion. All the above adaptations can promote cancer cell survival, stemness, and plasticity under stress conditions and ultimately contribute to therapy resistance (Fig. 2). However, one lacuna of many studies discussed herein is that they have been performed solely on a limited number of cell lines or patient samples. Complementary studies involving larger cohorts of primary specimens or PDXs are needed to validate and expand upon these findings, thus establishing the broader clinical relevance.

Our review also outlines and highlights the heterogeneity in mitochondrial phenotypes across different blood cancer types, subtypes, and clonal populations, such as LSCs versus blast cells. This diversity, coupled with the inherent genetic and molecular heterogeneity within leukemia and lymphoma subgroups, further complicates the comprehensive understanding of mitochondrial dynamics and the design of precision medicine targeting these pathways. At the same time, these suggest the need to further classify blood malignancies based on their mitochondrial status. In addition, while new single-cell technologies unravel the inter- and intra-patient clonal molecular heterogeneity in hematologic malignancies [204–206], they might fail to capture critical aspects of mitochondrial morphology because it is majorly controlled at the post-transcriptional level. High-resolution microscopy coupled with functional mitochondrial assays are essential to gain accurate and actionable insights.

Despite some progress in identifying the signaling pathways that converge and control mitochondrial morphology, the transcriptional, post-transcriptional, and post-translational regulators of mitochondrial dynamics in normal and malignant hematopoietic cell populations are still not completely mapped. While studies hint at AMPK and PI3/Akt signaling pathways controlling mitochondrial morphology and functions in solid tumors, their precise roles in blood cancers are still uncertain. Moreover, additional research is required to dissect how different genetic and epigenetic drivers of hematologic malignancies influence mitochondrial structure, dynamicity, and functions.

Interestingly, accumulating pieces of evidence hint that novel targeted anti-cancer drugs may modulate mitochondrial dynamics, either directly or indirectly. This consideration should be integrated into future preclinical evaluations for new therapies, as they might affect the drugs’ effectiveness. Mitochondrial dynamics proteins are increasingly recognized as potential therapeutic targets in various cancers, including specific blood malignancies, and in overcoming drug resistance. Recent medicinal chemistry efforts have resulted in the synthesis of lead compounds that block fusion or fission regulators. These include Mdivi-1, which has been tested in preclinical leukemia models with promise [110, 112, 125]. However, it is now known that Mdivi-1 inhibits not only DRP1 but also mitochondrial complex I, raising concerns about specificity and toxicities [207]. At commonly used concentrations, Mdivi-1 also lowers complex-I dependent ROS, indicating DRP1-independent actions that complicate result interpretation. Secondly, toxicity is dose- and cell-type-dependent as Mdivi-1 causes mitochondrial depolarization, oxidative stress, and apoptosis in oligodendrocytes under excitotoxic stress when under the commonly used dose range. The effects of Mdivi-1 on mitochondrial quality control can be double-edged. In ischemia or reperfusion models, Mdivi-1 reduces mitophagy markers alongside fission. Lastly, translation is hampered by heterogeneous dosing protocols, lack of side-effects, pharmacokinetics data, optimal administration parameters, and study-quality gaps. All of these make conclusions provisional and preclude clinical application at present [208]. More recently, novel OPA1 and MFN1/2 inhibitors have demonstrated potency and excellent results in combinatorial treatments tackling AML in preclinical models [127, 135, 137, 138, 155, 175]. Despite the promise, these compounds require further optimization to improve their solubility and bioavailability for effective in vivo testing and clinical translation. Besides, in malignancies such as AML, wherein the role of both fusion and fission modulators has been better characterized, further studies are required to explore fusion and fission combinatorial therapies addressing dual dependency. Based on the mitochondrial morphology state, can the dynamic timing of drugs administered restore the fusion-fission imbalance observed in disease?

**Fig. 3:**
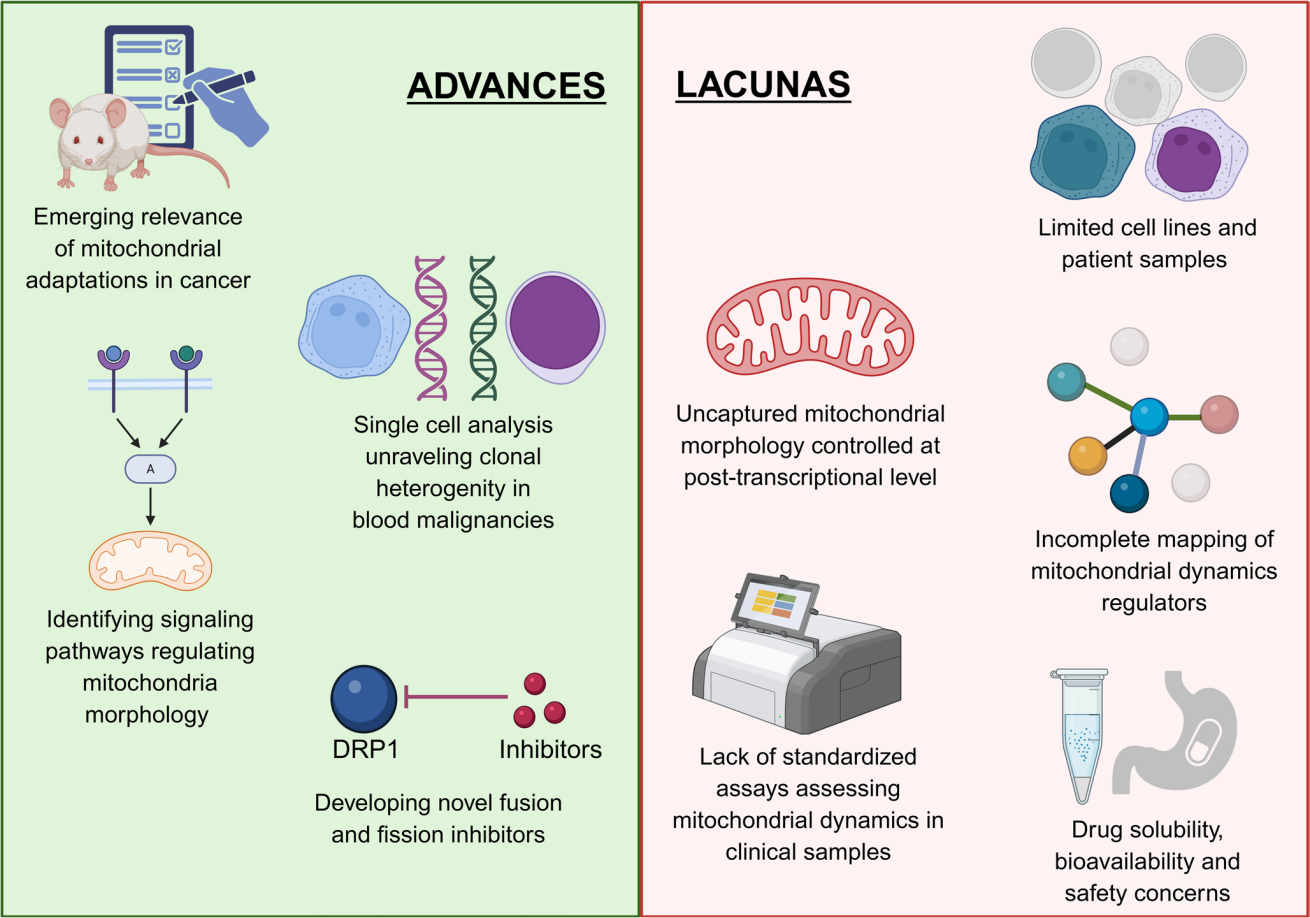
Advances vs. Lacunas. The studies on mitochondrial dynamics so far have provided deep insights into mitochondrial morphology pathways and the clonal heterogeneity in different blood cancers. With the established and upcoming blood cancer models and technologies, there is a significant scope for targeting mitochondrial dynamics in cancer therapeutics. Simultaneously, there is a need for new tools, standardized clinical assays, drug bioavailability and safety studies, and additional preclinical models along with improved fundamental understanding to determine the role of mitochondrial fission and fusion in blood malignancies (Figure was created in https://BioRender.com)

Lastly, our review reinforces the adaptive role of mitochondrial dynamics in the development of resistance to chemotherapy, irradiation, or targeted therapy in various leukemias or lymphomas. Hence, fusion and fission proteins, along with their upstream regulators, may serve as valuable biomarkers of therapy responsiveness in several conditions. Yet, appropriate and standardized assays for assessing mitochondrial dynamics in clinical samples need to be developed and validated. Figure 3 summarizes the advancements and lacunas in research on mitochondrial dynamics in cancer.

In conclusion, elucidating the contribution of mitochondrial dynamics in blood cancers presents new opportunities for rationally devising innovative therapeutic options, facilitating precision medicine strategies, and predicting treatment outcomes to ultimately improve patient care and survival.

## Outstanding Questions

The current understanding summarized in this review brings out several critical gaps in knowledge in the context of mitochondrial dynamics in hematologic malignancies. Addressing these questions is essential for translating basic mechanistic insights into therapeutic advances.

6.1 How are mitochondrial dynamics regulated across distinct blood cancer subtypes?
6.2 Do genetic and epigenetic drivers alter mitochondrial morphology and adaptations in hematologic neoplasms?
6.3 How do mitochondrial dynamics influence oncogenic signaling pathways and cellular stress responses in hematological malignancies?
6.4 How can heterogeneity in mitochondrial architecture and functions between blood cancer types be addressed in drug development?
6.5 With the development of mitochondrial dynamics-targeted drugs on the verge, what is their clinical translational potential?
6.6 Can mitochondrial dynamics inhibitors be effectively paired with conventional and emerging therapies synergistically or sequentially?
6.7 Can mitochondrial dynamics regulators represent new biomarkers for precision medicine?

## Key References

- Suzuki K., et al. BCR::ABL1-induced mitochondrial morphological alterations as a potential clinical biomarker in chronic myeloid leukemia*.* Cancer Sci. 2025; 116(3): p. 673-689.
  - This recent study is the first to highlight the role of *BCR-ABL1*-induced excessive mitochondrial fragmentation in CML proliferation.
- Glytsou C., et al. Mitophagy Promotes Resistance to BH3 Mimetics in Acute Myeloid Leukemia*.* Cancer Discov. 2023; 13(7): p. 1656-1677.
  - This study determines the potential of targeting mitochondrial dynamics-mediated quality control to overcome therapy resistance in AML.
- Hou D., et al. Interleukin-6 Facilitates Acute Myeloid Leukemia Chemoresistance via Mitofusin 1-Mediated Mitochondrial Fusion. Mol. Cancer Res. 2023; 21(12): p. 1366-1378.
  - This study unravels that IL6 induces chemoresistance in AML cells using increased mitochondrial fusion and OXPHOS dependency.
- Larrue C., et al. Mitochondrial fusion is a therapeutic vulnerability of acute myeloid leukemia*.* Leukemia 2023; 37(4): p. 765-775.
  - The study reports on metabolic and cell cycle impairment as consequences of targeting mitochondrial fusion mediators in AML.
- Decker C.W., et al. Mitofusin-2 mediates doxorubicin sensitivity and acute resistance in Jurkat leukemia cells*.* Biochem Biophys Rep 2020; 24: p. 100824.
  - The article explores the role of mitochondrial fusion in T-ALL cell survival and drug resistance.
- Chen X., et al. Targeting Mitochondrial Structure Sensitizes Acute Myeloid Leukemia to Venetoclax Treatment. Cancer Discov. 2019; 9(7): p. 890-909.
  - The study reports the influence of CLPB protein on mitochondrial architecture maintenance and its targetability in AML.
- Pei S., et al. AMPK/FIS1-Mediated Mitophagy Is Required for Self-Renewal of Human AML Stem Cells*.* Cell Stem Cell 2018; 23(1): p. 86-100 e6.
  - This study is the first to provide evidence about the roles of mitochondrial fission in stemness maintenance and quality control in AML LSCs.
- Cai J., et al. ERK/Drp1-dependent mitochondrial fission is involved in the MSC-induced drug resistance of T-cell acute lymphoblastic leukemia cells*.* Cell Death Dis. 2016; 7(11): p. e2459.
  - This is the first study to highlight that mitochondrial fission triggered by MSCs induces a pro-survival metabolic reprogramming in T-ALL.

## Data Availability

No datasets were generated or analysed during the current study.
